# External Validation of the Predictive Accuracy of Clinical and Immunological Scores in COVID-19 Outcomes in a Retrospective Cohort Study

**DOI:** 10.3390/biomedicines12112495

**Published:** 2024-10-31

**Authors:** Alina Doina Tanase, Emanuela-Lidia Petrescu, Teodora Hoinoiu, Daliana-Emanuela Bojoga, Bogdan Timar

**Affiliations:** 1 Department of Professional Legislation in Dental Medicine, Faculty of Dental Medicine, “Victor Babes” University of Medicine and Pharmacy, Eftimie Murgu Square No. 2, 300041 Timisoara, Romania; tanase.alina@umft.ro; 2 Doctoral School, “Victor Babes” University of Medicine and Pharmacy, Eftimie Murgu Square 2, 300041 Timisoara, Romania; 3 Department of Prostheses Technology and Dental Materials, Faculty of Dental Medicine, “Victor Babes” University of Medicine and Pharmacy, Eftimie Murgu Square 2, 300041 Timisoara, Romania; 4 Research Centre in Dental Medicine Using Conventional and Alternative Technologies, Faculty of Dental Medicine, “Victor Babes“ University of Medicine and Pharmacy, Eftimie Murgu Square 2, 300041 Timisoara, Romania; 5 Department of Clinical Practical Skills, “Victor Babes” University of Medicine and Pharmacy, Eftimie Murgu Square 2, 300041 Timisoara, Romania; tstoichitoiu@umft.ro; 6 Department of Oral Rehabilitation and Emergencies in Dental Medicine, Faculty of Dental Medicine, “Victor Babes” University of Medicine and Pharmacy, Eftimie Murgu Square 2, 300041 Timisoara, Romania; mocuta.daliana@umft.ro; 7 Interdisciplinary Research Canter for Dental Medical Research, Lasers, and Innovative Technologies, Faculty of Dental Medicine, “Victor Babes” University of Medicine and Pharmacy, Eftimie Murgu Square 2, 300041 Timisoara, Romania; 8 Second Department of Internal Medicine, “Victor Babes” University of Medicine and Pharmacy, Eftimie Murgu Square 2, 300041 Timisoara, Romania; bogdan.timar@umft.ro; 9 Centre for Molecular Research in Nephrology and Vascular Disease, “Victor Babes” University of Medicine and Pharmacy, Eftimie Murgu Square 2, 300041 Timisoara, Romania; 10 Department of Diabetes, “Pius Brinzeu” Emergency Hospital, 300723 Timisoara, Romania

**Keywords:** SARS-CoV-2, mortality, COVID-19, intensive care

## Abstract

Background and Objectives: The COVID-19 pandemic has necessitated the development of reliable prognostic tools to predict patient outcomes and guide clinical decisions. This study evaluates the predictive utility of several clinical scores—PAINT, ISARIC4C, CHIS, COVID-GRAM, SOFA, and CURB-65—for in-hospital mortality among COVID-19 patients, comparing their effectiveness at admission and seven days post-symptom onset. Methods: In this retrospective cohort study conducted at the Clinical Emergency Hospital Pius Brînzeu in Timișoara, adult patients hospitalized with confirmed SARS-CoV-2 infection were included. The study was approved by the Local Ethics Committee, adhering to GDPR and other regulatory standards. Prognostic scores were calculated using patient data at admission and Day 7. Statistical analyses included ROC curves, Kaplan–Meier survival analysis, and multivariate Cox regression. Results: The study comprised 269 patients, with a notable distinction in outcomes between survivors and non-survivors. Non-survivors were older (mean age 62.12 years) and exhibited higher comorbidity rates, such as diabetes (55.56% vs. 31.06%) and cardiovascular diseases (48.15% vs. 29.81%). Prognostic scores were significantly higher among non-survivors at both time points, with PAINT and ISARIC4C showing particularly strong predictive performances. The AUROC for PAINT increased from 0.759 at admission to 0.811 by Day 7, while ISARIC4C demonstrated an AUROC of 0.776 at admission and 0.798 by Day 7. Multivariate Cox regression indicated that a PAINT score above 8.10 by Day 7 was associated with a hazard ratio (HR) of 4.9 (95% CI: 3.12–7.72) for mortality. Conclusions: The study confirms the strong predictive value of the PAINT, ISARIC4C, CHIS, COVID-GRAM, SOFA, and CURB-65 scores in determining mortality risk among hospitalized COVID-19 patients. These scores can significantly aid clinicians in early-risk stratification and resource prioritization, potentially enhancing patient management and outcomes in acute care settings.

## 1. Introduction

The COVID-19 pandemic has exerted unprecedented pressure on global healthcare systems, emphasizing the critical need for accurate and reliable prognostic tools to predict patient outcomes [1,2,3]. By the end of 2023, the pandemic had resulted in over 800 million confirmed cases and more than 7 million deaths worldwide, straining healthcare resources and impacting economies globally [4,5,6]. Early identification of patients at high risk of severe disease progression or mortality is essential for optimizing clinical management and allocating limited resources effectively [7,8].

Several prognostic scoring systems have been developed to predict the severity and mortality risk in COVID-19 patients. Among these, the PAINT, ISARIC4C, CHIS, and COVID-GRAM scores have shown promise in initial studies [9,10]. The PAINT score incorporates pulmonary disease, age, immunoglobulin M (IgM) levels, natural killer (NK) cell counts, and aspartate aminotransferase (AST) levels [11]. The ISARIC4C Mortality Score includes age, sex, comorbidities, respiratory rate, oxygen saturation, Glasgow Coma Scale, blood urea nitrogen, and C-reactive protein [12]. The CHIS score evaluates hyperinflammatory syndrome features [13,14], while the COVID-GRAM score predicts severe outcomes based on clinical and laboratory parameters [15].

The Sequential Organ Failure Assessment (SOFA) score and the CURB-65 score are widely used prognostic tools in critical care and pneumonia management, respectively [16,17]. The SOFA score assesses the extent of a patient’s organ function or rate of failure, while the CURB-65 score evaluates the severity of pneumonia based on confusion, urea levels, respiratory rate, blood pressure, and age.

This study aims to validate the predictive utility of the PAINT, ISARIC4C, CHIS, and COVID-GRAM scores for mortality in a new cohort of hospitalized COVID-19 patients. Additionally, we compare these scores with the SOFA and CURB-65 scores to assess their relative performance in predicting mortality. We also aim to evaluate the predictive accuracy of these scores at admission and seven days post-symptom onset, and to analyze patient survival and ICU admission risk based on cutoffs expressed by these scores.

## 2. Materials and Methods

### 2.1. Legal and Ethical Considerations

In conducting this retrospective cohort study, several legal and ethical considerations were followed to ensure compliance with both national and European Union regulations concerning medical research. The study adhered strictly to the principles outlined in the Declaration of Helsinki, which provides a global framework for ethical conduct in human research, emphasizing informed consent, the right to privacy, and the obligation to mitigate potential harm.

Given the study’s retrospective nature, the Local Ethics Committee of the Clinical Emergency Hospital Pius Brînzeu in Timișoara reviewed and approved the research protocol (approval number 25/2023). The study period for retrospective data collection was spread between May 2020 and May 2023, ensuring to screen for patients who met the inclusion criteria and complete laboratory parameters to allow for the calculation of severity scores. An ethical waiver for informed consent was granted, which is permissible under EU and local Romanian law for retrospective analyses where the data are anonymized and the study poses minimal risk to participants. This waiver facilitated the examination of existing records without compromising patient confidentiality or autonomy.

The European Union has stringent regulations regarding medical research, primarily governed by the General Data Protection Regulation (GDPR) and the Clinical Trials Regulation (EU) No 536/2014. GDPR emphasizes the protection of personal data and privacy, requiring that all patient information be de-identified prior to analysis to prevent unauthorized access or identification. Our study’s compliance with GDPR was achieved through robust data anonymization processes, ensuring that individual patients could not be identified from the data used.

The Clinical Trials Regulation, on the other hand, provides a specific legal framework for conducting clinical trials on medicinal products across the EU. This regulation outlines the responsibilities regarding the safety, reporting, and transparency of clinical trials, which must be registered in a publicly accessible database. Although our study was observational and did not involve experimental treatments, awareness of these regulations was essential to uphold the ethical standards and integrity of the research.

Special attention was given to data protection and confidentiality, which are critical in all medical research but especially in studies involving sensitive health data. All electronic medical records were accessed only by authorized personnel, and identifiers were removed before the dataset was analyzed. These measures comply with both local data protection laws and EU-wide directives that safeguard patient information.

The ethical obligation extends to the dissemination of the study findings. Publications resulting from the study will need to ensure that the data remain anonymized, and that interpretations and conclusions drawn from the data are conducted responsibly, without causing harm to the subjects from whom the data were derived. The publication will also adhere to the guidelines for ethical reporting outlined by major medical journals, which include declarations of any conflicts of interest and funding sources that could influence the study’s outcomes.

Finally, an ongoing commitment to monitoring compliance with both ethical and legal standards was maintained throughout the study period. This included regular audits of data use and security, as well as continuous updates to the research team about changes in legislation or ethical guidelines related to medical research in the European Union. This proactive approach ensures that the study upholds the highest standards of ethical practice and legal compliance, reflecting the evolving nature of both medical research and regulatory landscapes.

### 2.2. Inclusion and Exclusion Criteria

We included adult patients (≥18 years) who were hospitalized with confirmed SARS-CoV-2 infection, verified using reverse transcription-polymerase chain reaction (RT-PCR) testing. Eligible patients had the complete clinical and laboratory data required to calculate the PAINT, ISARIC4C, CHIS, COVID-GRAM, SOFA, and CURB-65 scores at admission and seven days after symptom onset. Exclusion criteria included patients transferred from other hospitals without initial scoring data, patients discharged or deceased before the seven-day evaluation, and patients with incomplete records missing essential clinical parameters or outcomes.

### 2.3. Study Variables

Data were extracted from electronic medical records and included demographic information (age, sex), comorbidities (including diabetes mellitus, hypertension, cardiovascular disease, chronic lung disease), clinical parameters (respiratory rate, oxygen saturation, Glasgow Coma Scale, systolic blood pressure, heart rate, temperature), and laboratory values (white blood cell count, lymphocyte count, AST, blood urea nitrogen, C-reactive protein, D-dimer, ferritin, interleukin-6, creatinine, bilirubin, platelet count). Data were collected at two time points, at admission and seven days after symptom onset.

### 2.4. Definitions

The PAINT, ISARIC4C, CHIS, COVID-GRAM, SOFA, and CURB-65 scores were calculated for each patient at both time points using the published scoring systems [9,10,11,12,14,15]. The PAINT score includes pulmonary disease, age > 75 years, IgM levels < 0.84 mg/dL, NK cell counts < 116.5 cells/μL, and AST > 25 U/L. The ISARIC4C Mortality Score includes age, sex, comorbidities, respiratory rate, oxygen saturation, Glasgow Coma Scale, blood urea nitrogen, and C-reactive protein. The CHIS score evaluates fever, macrophage activation, hematological dysfunction, hepatic inflammation, coagulopathy, and elevated cytokine levels. The COVID-GRAM score includes age, respiratory symptoms, comorbidities, lymphocyte count, and C-reactive protein. The SOFA score assesses organ dysfunction based on respiratory, cardiovascular, hepatic, coagulation, renal, and neurological systems. The CURB-65 score includes confusion, urea > 7 mmol/L, respiratory rate ≥ 30 breaths/min, systolic blood pressure < 90 mmHg or diastolic ≤ 60 mmHg, and age ≥ 65 years.

The primary outcome was in-hospital mortality. Secondary outcomes included intensive care unit (ICU) admission, need for mechanical ventilation, and length of hospital stay.

During the COVID-19 pandemic in Romania, therapeutic approaches were adapted to manage and treat patients based on disease severity. For those with severe cases, treatment often included ICU admission and mechanical ventilation. The therapeutic protocols typically featured antiviral agents such as Remdesivir, particularly for early-stage viral replication reduction. Azithromycin was used, albeit cautiously, for its potential anti-inflammatory effects and prophylaxis against secondary bacterial infections, despite mixed evidence regarding its efficacy against COVID-19 specifically. Corticosteroids like dexamethasone became standard for reducing inflammation in severe cases. Supportive treatments were also crucial and included oxygen therapy, fluid management, and, in extreme cases, extracorporeal membrane oxygenation (ECMO) for critically ill patients.

### 2.5. Statistical Analysis

Statistical analyses were performed using SPSS Statistics version 26.0 (IBM Corp., Armonk, NY, USA). Continuous variables were expressed as mean ± standard deviation (SD), and categorical variables as frequencies and percentages. Comparisons between survivors and non-survivors were made using Student’s *t*-test for continuous variables and a Chi-square test or Fisher’s exact test for comparing proportions.

Receiver operating characteristic (ROC) curves were generated to assess the predictive accuracy of each score for mortality, and the area under the curve (AUC) was calculated. Optimal cutoff values were determined using the Youden index. Sensitivity, specificity, positive predictive value (PPV), and negative predictive value (NPV) were calculated. Kaplan–Meier survival curves were generated to compare survival times between groups stratified by the optimal cutoff values of each score, and the log-rank test was used to assess differences between survival curves. Multivariate Cox proportional hazards regression analysis was performed to identify independent predictors of mortality, adjusting for potential confounders such as age, sex, and comorbidities. Hazard ratios (HR) and 95% confidence intervals (CI) were reported. A *p*-value < 0.05 was considered statistically significant.

## 3. Results

It was observed that non-survivors were, on average, older than survivors, with a mean age of 62.12 years compared to 58.45 years. Non-survivors also had a higher mean BMI of 29.98 kg/m^2^, while survivors had a mean BMI of 28.47 kg/m^2^. A notable difference was seen in the prevalence of diabetes mellitus, present in 55.56% of non-survivors versus 31.06% of survivors. Cardiovascular disease was also more common among non-survivors (48.15%) compared to survivors (29.81%). ICU admission and mechanical ventilation were significantly more frequent among non-survivors, with 69.44% requiring ICU admission and 64.81% needing mechanical ventilation, compared to only 9.94% and 4.97%, respectively, among survivors. Non-survivors had a longer mean hospital stay (15.04 days) compared to survivors (12.46 days), as seen in Table 1.

Non-survivors had a significantly lower mean SpO_2_ of 85.62% compared to 94.28% in survivors. The mean respiratory rate was markedly higher in non-survivors (27 breaths/min) than in survivors (18 breaths/min). Similarly, non-survivors exhibited a higher heart rate (103.52 bpm) versus survivors (88.29 bpm). Laboratory values revealed that non-survivors had elevated markers of inflammation and infection, with a mean white blood cell count of 11.34 × 10^9^/L, compared to 6.45 × 10^9^/L in survivors. Non-survivors also showed significantly higher C-reactive protein (CRP) levels (156.45 mg/L) compared to survivors (20.85 mg/L). Additionally, mean D-dimer levels were markedly elevated in non-survivors (3.97 mg/L) versus survivors (0.56 mg/L). Prognostic scores, such as the PAINT, ISARIC4C, CHIS, COVID-GRAM, SOFA, and CURB-65, were substantially higher in non-survivors, reflecting a higher disease severity (Table 2).

The comparison of clinical prediction scores revealed that non-survivors had significantly worse outcomes. The mean SpO_2_ was lower in non-survivors (84.40%) compared to survivors (95.20%). Non-survivors had a notably higher respiratory rate (28 breaths/min) versus survivors (17.30 breaths/min). Inflammatory markers such as WBC count (15.31 × 10^9^/L) and CRP levels (180.85 mg/L) were also much higher in non-survivors. Prognostic scores, including PAINT (9.02 vs. 3.52), ISARIC4C (14.05 vs. 4.02), and SOFA (8.05 vs. 1.52), were significantly elevated in non-survivors, indicating their predictive value in determining patient outcomes (Table 3).

The AUROC analysis demonstrated that all clinical scores showed strong predictive values for mortality, both at admission and at Day 7. For the PAINT score, a cutoff of 6.28 at admission resulted in a sensitivity of 85.56% and a specificity of 77.02% (AUC = 0.759), which improved by Day 7, where a cutoff of 8.10 increased sensitivity to 90.28% and specificity to 79.5% (AUC = 0.811). The ISARIC4C score also demonstrated high predictive accuracy, with an AUC of 0.776 at admission and 0.798 by Day 7. The CHIS score showed a notable improvement from an AUC of 0.641 at admission to 0.885 by Day 7. Similarly, other scores, including COVID-GRAM, SOFA, and CURB-65, maintained strong predictive capacities over time, with incremental improvements from admission to Day 7 (Table 4).

**Figure 1:**
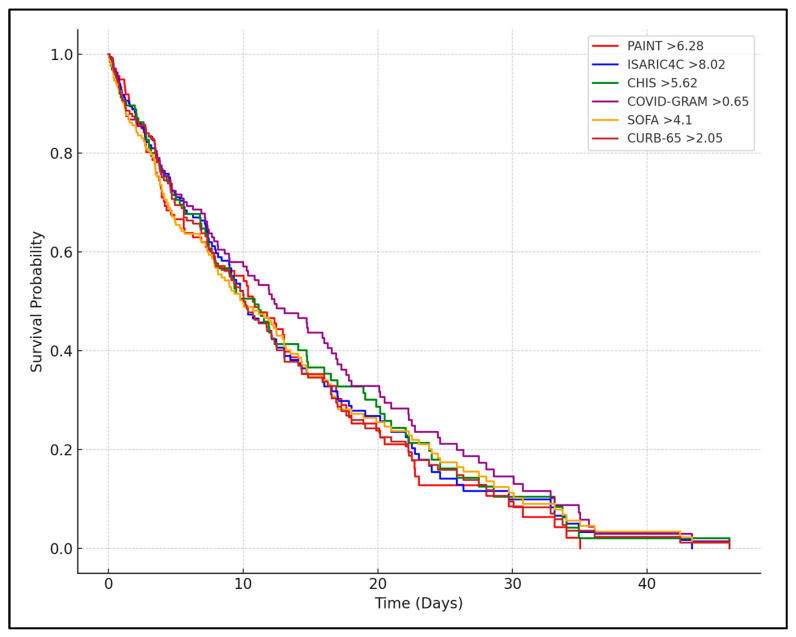
Kaplan–Meier analysis for mortality.

In the multivariate Cox regression analysis, it was observed that higher clinical scores at admission and Day 7 were strongly associated with increased mortality risk. For example, a PAINT score greater than 6.28 at admission was associated with a hazard ratio (HR) of 3.5 (95% CI: 2.15–5.79), which increased to 4.9 (95% CI: 3.12–7.72) by Day 7 for scores above 8.10. Similar trends were noted for the ISARIC4C score, where a cutoff of 8.02 at admission yielded an HR of 2.9, and a Day 7 cutoff of 9.19 raised the HR to 3.7. Other scores, such as CHIS, COVID-GRAM, and SOFA, also showed significantly elevated mortality risks with increasing scores over time, reinforcing their utility as predictors of death (Table 5 and Figure 1).

**Figure 2:**
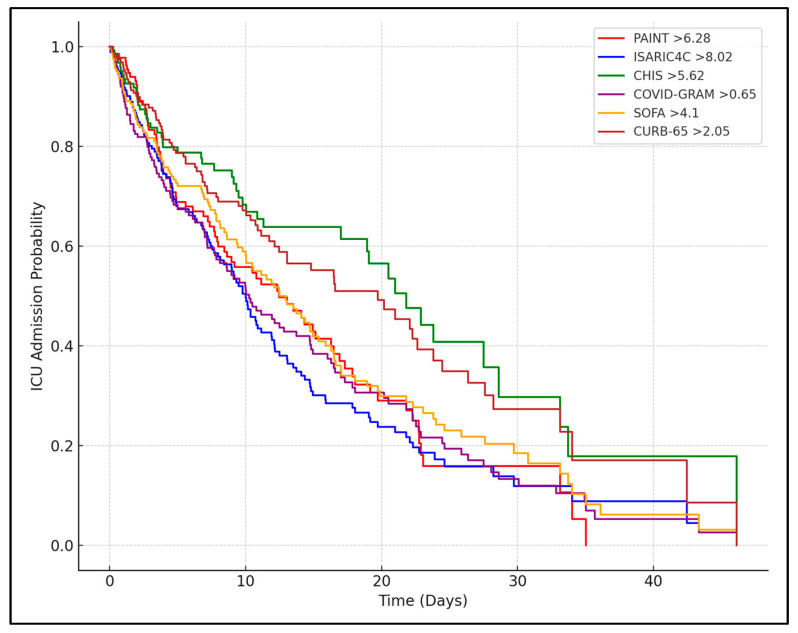
Kaplan–Meier curve for ICU admissions.

The Cox regression analysis for ICU admission indicated that higher clinical scores at both admission and Day 7 were predictive of ICU admission. At admission, an ISARIC4C score greater than 8.87 was associated with a hazard ratio of 3.5 (95% CI: 2.87–4.28), and the CHIS score had an HR of 4.1 for a cutoff of 5.94. By Day 7, the PAINT score had a hazard ratio of 5.2 for values above 8.39, indicating a substantial increase in the likelihood of ICU admission. Other scores, including the ISARIC4C, CHIS, and COVID-GRAM, showed similarly strong associations with ICU admission, particularly by Day 7, where their predictive power further increased (Table 6 and Figure 2).

## 4. Discussion

### 4.1. Analysis of Findings

The study demonstrated a robust predictive value of various clinical scores for mortality among hospitalized COVID-19 patients. Notably, prognostic scores like PAINT, ISARIC4C, CHIS, COVID-GRAM, SOFA, and CURB-65 were significantly higher among non-survivors, emphasizing their utility in predicting patient outcomes. The multivariate Cox regression analysis reinforced this finding, illustrating that higher scores at both admission and Day 7 were strongly associated with increased mortality risk. For instance, a PAINT score greater than 6.28 at admission correlated with a hazard ratio of 3.5, which escalated to 4.9 for scores above 8.10 by Day 7. This pattern was consistent across other scores, indicating their effectiveness in early-risk stratification and ongoing patient monitoring.

The clinical utility of these findings is substantial, offering healthcare providers a reliable toolset for the early identification of patients at high risk of mortality. By effectively stratifying patients based on their prognostic scores at admission and monitoring their progression through Day 7, clinicians can prioritize resources, adjust treatment plans, and potentially improve outcomes by intervening more aggressively in high-risk cases. This could be particularly valuable in settings with limited resources where efficient patient management is important.

In a similar manner, the study by Gupta et al. [18] developed and validated the ISARIC4C Deterioration model, aiming to predict clinical deterioration in hospitalized COVID-19 patients. Their findings from over 74,944 participants revealed a significant clinical deterioration rate of 43.2%, demonstrating the model’s consistency across diverse regions with a C-statistic of 0.77 and calibration slope of 0.96 in the London validation cohort. This robust performance highlights the model’s potential for widespread clinical application in predicting patient outcomes at the point of hospital admission. Similarly, Crocker-Buque et al. [19] explored the dynamic application of the ISARIC4C mortality score, assessing changes in mortality risk during hospital stays. Their analysis of 6373 patients showed an improvement in the predictive accuracy of the mortality score from an AUC of 0.71 at admission to 0.82 by Day 7, indicating the score’s enhanced reliability over the hospitalization period.

Moreover, the study by Vallipuram et al. [20] sought to externally validate the ISARIC4C Mortality Score among critically ill COVID-19 patients in a Canadian ICU, comparing its performance against established scores like APACHE II and SOFA. Their results indicated that the ISARIC4C score, with an area under the curve (AUC) of 0.762, outperformed both the SOFA and APACHE II scores, which posted AUCs of 0.705 and 0.722, respectively, thus affirming the utility of the ISARIC4C score in predicting in-hospital mortality. This finding echoes the study by Doğanay et al. [21], where the ISARIC4C score was also compared alongside other scoring systems such as CURB-65 and COVID-GRAM for predicting in-hospital mortality and ICU requirements in COVID-19 patients. Although the ISARIC4C score had an AUC of 0.784 for mortality, which was lower than the CURB-65’s AUC of 0.846, it still demonstrated reliable predictive power, particularly in identifying patients at lower risk.

Other research explored the efficacy of the COVID-GRAM score in stratifying COVID-19 patients into low-, medium-, and high-risk categories for complications and mortality, based on a large cohort from the COLOS registry [15]. They found that patients in the high-risk category, constituting 13% of the study population, demonstrated significantly higher comorbidity rates and were more likely to suffer severe outcomes such as requiring dialysis or increased mortality. The area under the curve for the COVID-GRAM score suggested its effectiveness in predicting these outcomes. Similarly, Armiñanzas et al. [22] assessed the usefulness of the COVID-GRAM and CURB-65 scores in predicting the severity of COVID-19 and found that a high COVID-GRAM score was strongly predictive of critical illness, with an odds ratio of 9.40 and an AUC of 0.779. This was comparable to CURB-65, which also demonstrated strong predictive performance with an AUC of 0.83 for 30-day mortality.

In a similar manner, the study by Lombardi et al. [23] sought to evaluate the efficacy of multiple prognostic scores for predicting in-hospital mortality and ICU transfers among COVID-19 patients in the Greater Paris University Hospitals. Their comprehensive analysis of 14,343 patients revealed that 35% either died or were transferred to the ICU. The study tested various scores and found that seven had an area under the curve (AUC) greater than 0.75 for predicting in-hospital mortality, suggesting a fairly good predictive performance. This mirrors the findings of Bradley et al. [24], who assessed the effectiveness of the Pneumonia Severity Index (PSI) and CURB-65 score in predicting mortality among patients with SARS-CoV-2 community-acquired pneumonia (CAP). Their study, involving two prospective cohorts, showed that PSI and CURB-65 had AUCs of 0.82 and 0.79, respectively, for SARS-CoV-2 CAP, indicating strong prognostic capabilities.

### 4.2. Study Limitations

The study exhibited several limitations that could impact the generalizability and robustness of its findings. Conducted at a single hospital, the sample homogeneity limited its applicability to broader populations, as it may not have reflected the demographic and healthcare diversity of other regions. Its retrospective design introduced potential biases, from inconsistent record-keeping to variations in diagnostic practices, which could have affected data accuracy. Moreover, assessing patient outcomes at only two time points—admission and seven days post-symptom onset—may have omitted crucial changes in patient status that occurred at other stages of the disease progression. Additionally, the lack of comparative data from other hospitals or regions prevented validation of these findings across different healthcare contexts, hindering the ability to confirm their wider applicability. Moreover, the study’s reliance on clinical data such as respiratory rate, SpO_2_, and WBC counts, which require timely and accurate measurement, may not be feasible in all clinical settings. This limitation could affect the generalizability and application of the findings in environments with varied medical data collection capabilities.

## 5. Conclusions

In conclusion, this study validates the effectiveness of PAINT, ISARIC4C, CHIS, COVID-GRAM, SOFA, and CURB-65 scores as predictors of mortality in COVID-19 patients, supporting their use as vital tools in clinical decision-making. These findings underscore the importance of integrating clinical scoring systems into the routine management of COVID-19 to enhance patient care and optimize clinical outcomes.

## Figures and Tables

**Table 1:** Demographic and clinical characteristics by survival status.

Variables	Survivors (*n* = 161)	Non-Survivors (*n* = 108)	*p*
Age, years (mean ± SD)	58.45 ± 15.32	62.12 ± 13.56	0.041
Male gender, *n* (%)	88 (54.66%)	64 (59.26%)	0.438
BMI (kg/m^2^, mean ± SD)	28.47 ± 5.23	29.98 ± 5.84	0.029
Hypertension, *n* (%)	91 (56.52%)	71 (65.74%)	0.112
Diabetes mellitus, *n* (%)	50 (31.06%)	60 (55.56%)	<0.001
Cardiovascular disease, *n* (%)	48 (29.81%)	52 (48.15%)	0.003
Chronic lung disease, *n* (%)	28 (17.39%)	26 (24.07%)	0.168
Vaccinated, *n* (%)	31 (19.25%)	13 (12.04%)	0.116
ICU admission, *n* (%)	16 (9.94%)	75 (69.44%)	<0.001
Mechanical ventilation, *n* (%)	8 (4.97%)	70 (64.81%)	<0.001
Length of hospital stay (days)	12.46 ± 6.02	15.04 ± 7.21	0.003

BMI—Body Mass Index; ICU—Intensive Care Unit.

**Table 2:** Baseline clinical and laboratory values by survival status at admission.

Variables (Mean ± SD)	Survivors (*n* = 161)	Non-Survivors (*n* = 108)	*p*
SpO_2_ (%)	94.28 ± 1.78	85.62 ± 3.31	<0.001
Respiratory rate (breaths/min)	18.52 ± 2.08	27.28 ± 5.82	<0.001
Heart rate (bpm)	88.29 ± 12.38	103.52 ± 19.18	<0.001
Temperature (°C)	37.12 ± 0.44	38.48 ± 0.80	<0.001
WBC (×10^9^/L)	6.45 ± 1.36	11.34 ± 4.21	<0.001
Lymphocyte count (×10^9^/L)	1.46 ± 0.47	0.83 ± 0.36	<0.001
CRP (mg/L)	20.85 ± 10.42	156.45 ± 70.38	<0.001
Ferritin (ng/mL)	250.38 ± 110.82	743.12 ± 331.05	<0.001
IL-6 (pg/mL)	12.38 ± 4.65	46.95 ± 17.25	<0.001
D-dimer (mg/L FEU)	0.56 ± 0.24	3.97 ± 1.76	<0.001
PAINT score	3.24 ± 1.10	7.86 ± 2.58	<0.001
ISARIC4C score	4.24 ± 1.35	11.42 ± 3.65	<0.001
CHIS score	2.55 ± 1.23	8.36 ± 3.14	<0.001
COVID-GRAM score	0.33 ± 0.13	0.90 ± 0.24	<0.001
SOFA score	2.01 ± 1.02	6.02 ± 2.01	<0.001
CURB-65 score	1.01 ± 0.51	3.01 ± 1.02	<0.001

SD—Standard Deviation; WBC—White Blood Cell; ISARIC4C—International Severe Acute Respiratory and emerging Infection Consortium Coronavirus Clinical Characterization Consortium Mortality Score; CHIS—COVID-19-associated Hyperinflammatory Syndrome; COVID-GRAM—COVID-GRAM Risk Score.

**Table 3:** Clinical prediction score comparisons by survival status at 7 days.

Variables (Mean ± SD)	Survivors (*n* = 161)	Non-Survivors (*n* = 108)	*p*
SpO_2_ (%)	95.20 ± 1.90	84.40 ± 4.28	<0.001
Respiratory rate (breaths/min)	17.30 ± 3.15	28.72 ± 7.48	<0.001
WBC (×10^9^/L)	7.54 ± 2.05	15.31 ± 5.85	<0.001
Lymphocyte count (×10^9^/L)	1.66 ± 0.63	0.80 ± 0.31	<0.001
CRP (mg/L)	15.70 ± 6.48	180.85 ± 90.62	<0.001
PAINT score	3.52 ± 1.52	9.02 ± 3.02	<0.001
ISARIC4C score	4.02 ± 2.01	14.05 ± 4.98	<0.001
CHIS score	2.52 ± 1.02	11.02 ± 4.02	<0.001
COVID-GRAM score	0.36 ± 0.16	0.92 ± 0.31	<0.001
SOFA score	1.52 ± 0.52	8.05 ± 3.05	<0.001
CURB-65 score	0.52 ± 0.52	4.02 ± 1.02	<0.001

SD—Standard Deviation; WBC—White Blood Cell; ISARIC4C—International Severe Acute Respiratory and emerging Infection Consortium Coronavirus Clinical Characterization Consortium Mortality Score; CHIS—COVID-19-associated Hyperinflammatory Syndrome; COVID-GRAM—COVID-GRAM Risk Score.

**Table 4:** AUROC analysis of clinical prediction scores at admission and Day 7.

Parameters	Timeframe	Best Cutoff Value	Sensitivity	Specificity	AUC	*p*
PAINT	Admission	6.28	85.56	77.02	0.759	<0.001
	Day 7	8.1	90.28	79.5	0.811	<0.001
ISARIC4C	Admission	8.02	80.65	82.15	0.776	<0.001
	Day 7	9.12	87.96	81.62	0.798	<0.001
CHIS	Admission	5.62	88.89	74.85	0.641	<0.001
	Day 7	7.8	91.67	74.3	0.885	<0.001
COVID-GRAM	Admission	0.65	83.33	78.41	0.749	<0.001
	Day 7	0.7	85.65	80.31	0.692	<0.001
SOFA	Admission	4.1	82.22	77.65	0.852	<0.001
	Day 7	6.0	89.35	80.1	0.674	<0.001
CURB-65	Admission	2.05	79.82	76.12	0.843	<0.001
	Day 7	3.1	84.6	77.3	0.665	<0.001

**Table 5:** Multivariate Cox regression analysis for mortality risk based on measurements at 7 days.

Score	Time Point	Cutoff Value	Hazard Ratio	95% CI	*p*
PAINT	Admission	>6.28	3.5	2.15–5.79	0.001
	Day 7	>8.10	4.9	3.12–7.72	<0.001
ISARIC4C	Admission	>8.02	2.9	1.90–4.53	0.001
	Day 7	>9.19	3.7	2.35–5.80	<0.001
CHIS	Admission	>5.62	4.0	2.60–6.35	<0.001
	Day 7	>7.82	5.3	3.42–8.26	<0.001
COVID-GRAM	Admission	>0.66	3.15	2.05–4.95	<0.001
	Day 7	>0.70	4.25	2.70–6.72	<0.001
SOFA	Admission	>4.1	2.82	1.88–4.26	<0.001
	Day 7	>6.08	3.62	2.42–5.45	<0.001
CURB-65	Admission	>2.05	2.52	1.68–3.80	0.006
	Day 7	>3.13	3.22	2.12–4.92	<0.001

**Table 6:** Multivariate Cox regression analysis for ICU admission.

Score	Time Point	Cutoff Value	Hazard Ratio	95% CI	*p*
PAINT	Admission	6.13	2.1	0.93–2.47	0.083
ISARIC4C	Admission	8.87	3.5	2.87–4.28	<0.001
CHIS	Admission	5.94	4.1	3.54–4.68	<0.001
COVID-GRAM	Admission	0.61	2.8	2.14–3.58	<0.001
SOFA	Admission	4.21	3.0	2.24–3.84	<0.001
CURB-65	Admission	2.28	2.3	1.00–2.98	0.062
PAINT	Day 7	8.39	5.2	4.18–6.37	<0.001
ISARIC4C	Day 7	9.48	4.6	3.25–4.86	<0.001
CHIS	Day 7	7.91	5.5	4.34–6.63	<0.001
COVID-GRAM	Day 7	0.74	4.8	3.67–6.01	<0.001
SOFA	Day 7	6.18	3.6	2.83–4.47	0.001
CURB-65	Day 7	3.26	4.2	3.22–5.18	<0.001

## Data Availability

The data presented in this study are available on request from the corresponding author.
